# Circ_0061012 contributes to IL-22-induced proliferation, migration and invasion in keratinocytes through miR-194-5p/GAB1 axis in psoriasis

**DOI:** 10.1042/BSR20203130

**Published:** 2021-01-14

**Authors:** Qi He, Nian Liu, Feng Hu, Quan Shi, Xianming Pi, Hongxiang Chen, Jiawen Li, Bo Zhang

**Affiliations:** 1 Department of Dermatology, Hubei Provincial Hospital of Traditional Chinese Medicine, Hubei Province Academy of Traditional Chinese Medicine, Wuhan, Hubei, China; 2 Department of Dermatology, Union Hospital, Tongji Medical College, Huazhong University of Science and Technology, Wuhan, Hubei, China; 3 Department of Dermatology, Wuhan No. 1 Hospital, Tongji Medical College, Huazhong University of Science and Technology, Wuhan, Hubei, China

**Keywords:** circ_0061012, GAB1, IL-22, miR-194-5p, psoriasis

## Abstract

Psoriasis is a chronic inflammation-associated skin disorder featured by excessive proliferation and abnormal differentiation of keratinocytes. Here, we intended to investigate the role of circular RNA 0061012 (circ_0061012) in psoriasis progression. The expression of circ_0061012, SLMO2-ATP5E readthrough (SLMO2-ATP5E) messenger RNA (mRNA), microRNA-194-5p (miR-194-5p) and GRB2 associated binding protein 1 (GAB1) mRNA was determined by quantitative real-time polymerase chain reaction (qRT-PCR). Cell proliferation and metastasis were analyzed by 3-(4,5-Dimethylthiazol-2-yl)-2,5-diphenyltetrazolium bromide (MTT) assay and transwell assays. Western blot assay was used to measure the protein levels of Ki67, matrix metallopeptidase 9 (MMP9) and GAB1. Dual-luciferase reporter assay and RNA immune co-precipitation (RIP) assay were used to verify the interaction between miR-194-5p and circ_0061012 or GAB1. Circ_0061012 abundance was significantly enhanced in lesional skin samples from psoriasis patients than that in normal skin specimens from healthy volunteers. Interleukin-22 (IL-22) treatment increased the expression of circ_0061012 in a dose-dependent manner. Circ_0061012 silencing alleviated IL-22-induced promoting effects in the proliferation, migration and invasion of HaCaT cells. Circ_0061012 interacted with miR-194-5p, and miR-194-5p knockdown counteracted circ_0061012 silencing-mediated influences in IL-22-induced HaCaT cells. GAB1 was a target of miR-194-5p in HaCaT cells, and miR-194-5p hampered proliferation and metastasis which were induced by IL-22 partly through targeting GAB1. Circ_0061012 elevated the expression of GAB1 through sponging miR-194-5p in HaCaT cells. Circ_0061012 accelerated IL-22-induced proliferation and metastasis in HaCaT cells through enhancing GAB1 expression via sponging miR-194-5p in psoriasis.

## Introduction

Psoriasis is an autoimmune skin disorder characterized by hyper-proliferation, aberrant motility and differentiation of keratinocytes and the infiltration of inflammatory cells [1–3]. Immune, genetic and environmental factors are all responsible for the pathogenesis of psoriasis [4]. Pro-inflammatory cytokines secreted by immune cells promote the proliferation of keratinocytes [5]. Interleukin-22 (IL-22) belongs to IL-10 superfamily [6], and the messenger RNA (mRNA) level of IL-22 was positively correlated with the severity of psoriasis [7,8]. Here, we established psoriasis cell model through stimulating keratinocytes HaCaT with IL-22.

Compared with linear RNAs, circular RNAs (circRNAs) are stably distributed due to their covalently closed loop structure [9]. CircRNAs exert crucial functions in aging, tissue development, cancers and diseases [10]. Circular RNA 0061012 (Circ_0061012) was highly expressed in the lesional skin samples compared with healthy skin samples [11]. Nevertheless, the precise role of circ_0061012 in psoriasis progression remains to be discovered.

MicroRNAs (miRNAs) were found to modulate the development of psoriasis [12,13]. Numerous miRNAs were abnormally expressed in the lesional tissues of psoriasis patients [14,15]. Yu et al. proved that miR-194 overexpression hampered the proliferation while accelerated the differentiation of keratinocytes through GRHL2 in psoriasis [16]. Nevertheless, the exact role of miR-194-5p in psoriasis is not fully addressed.

GRB2 associated binding protein 1 (GAB1) acts as the docking protein to facilitate the growth and differentiation of epithelium [17]. GAB1 was observed to regulate the migration ability of keratinocytes, implying GAB1 might exert essential role in psoriasis progression [18]. Liu et al. claimed that miR-183-3p restrained the growth and migration abilities of HaCaT cells via inhibiting GAB1 in psoriasis [19]. We discovered the potential mechanism behind the role of GAB1 in psoriasis.

In the present study, the expression pattern and biological role of circ_0061012 in psoriasis were explored. Subsequently, the downstream targets of circ_0061012 were predicted by bioinformatics software to further illustrate the working mechanism of circ_0061012 in psoriasis progression.

## Materials and methods

### Clinical samples

Twenty-seven psoriasis patients along with twenty-seven healthy volunteers were enrolled in Hubei Provincial Hospital of Traditional Chinese Medicine. Four-millimeter punch biopsies were collected from psoriatic lesional skin tissues of psoriasis patients. Punch biopsies collected from the healthy skin tissues were utilized as control group. All cases had signed informed consents. The protocols were approved by the Ethic Committee of Hubei Provincial Hospital of Traditional Chinese Medicine.

### Cell culture

Keratinocyte cell line HaCaT was obtained from BeNa Culture Collection (Beijing, China) and maintained with Dulbecco’s modified Eagle’s medium (DMEM; Gibco, Carlsbad, CA, U.S.A.) containing 10% fetal bovine serum (FBS; Gibco) and 100 units/ml penicillin/100 μg/ml streptomycin solution (Sangon Biotech, Shanghai, China) with 5% CO_2_ at 37°C cell culture incubator.

### IL-22 stimulation

When cell confluence reached approximately 80%, HaCaT cells were pre-treated in serum-free medium for 24 h, and these cells were exposed to increased doses of IL-22 (Sangon Biotech) for an additional 24 h.

### Subcellular fractionation location and RNase R digestion

Cytoplasmic/nuclear RNA isolation was implemented with the PARIS™ Kit Protein and RNA Isolation system (Thermo Fisher Scientific, Waltham, MA, U.S.A.). Cell supernatant was utilized to measure the levels of cytoplasmic RNAs, and nuclear pellet was collected to determine the levels of nuclear RNAs. U6 small nuclear RNA and glyceraldehyde-3-phosphate dehydrogenase (GAPDH) were utilized as nuclear endogenous control and cytoplasmic endogenous control, respectively. RNA levels were determined by quantitative reverse transcription polymerase chain reaction (qRT-PCR). The distribution ratio of cytoplasm to nucleus was analyzed through dividing the expression of circ_0061012 in cytoplasmic fraction by the expression of circ_0061012 in nuclear fraction.

Total RNA was digested with 3 U/μg RNase R (Epicentre Technologies, Madison, WI, U.S.A.) for 40 min at room temperature. RNA levels were measured via qRT-PCR.

### qRT-PCR

To measure the enrichment of circ_0061012, SLMO2-ATP5E readthrough (SLMO2-ATP5E) mRNA and GAB1 mRNA, moloney murine leukemia virus reverse transcriptase (Takara, Dalian, China) was used to synthesize complementary DNA (cDNA). The reverse transcription of miR-194-5p was conducted using the TaqMan microRNA reverse transcription kit (Applied Biosystems, Carlsbad, CA, U.S.A.). SYBR Green Master Mix Kit (Bio-Rad, Hercules, CA, U.S.A.) and primers (shown in Table 1) were used for amplification reaction. The RNA expression was analyzed using the 2^−ΔΔ*C*_t_^ method. GAPDH served as the internal control for circ_0061012, SLMO2-ATP5E mRNA and GAB1 mRNA, while miR-194-5p enrichment was normalized to U6.

**Table 1:** Primers in qRT-PCR

Gene	Direction	Forward primer	Reverse primer
*circ_0061012*	5′–3′	TACTCTTGAGGCCGAGAAGC	GCTGCCATAGTCCTTAT
*SLMO2-ATP5E*	5′–3′	TGCAGAAATACCCAAACCCT	AATGTTCTTGCACATATGTT
*miR-194-5p*	5′–3′	GCGGCGGTGTAACAGCAACTCC	ATCCAGTGCAGGGTCCGAGG
*GAB1*	5′–3′	ATGAGCGGCGGCGAAGTGGTTTGCT	CGCGACTGAAGAAGCTTCCATCTGA
*U6*	5′–3′	GCTTCGGCAGCACATATACTA	CGCTTCACGAATTTGCGTGTC
*GAPDH*	5′–3′	TATGATGATATCAAGAGGGTAGT	TGTATCCAAACTCATTGTCATAC

### Cell transfection

Circ_0061012 specific small interfering RNA (si-circ_0061012) and scramble siRNA negative control (si-NC) were purchased from Dharmacon (Lafayette, CO, U.S.A.). Circ_0061012 overexpression plasmid (circ_0061012) and pLCDH-cir (Vector) were purchased from Ribobio (Guangzhou, China). MiR-194-5p mimics (miR-194-5p), miR-NC, miR-194-5p inhibitor (anti-miR-194-5p), anti-miR-NC, GAB1 plasmid (GAB1) and pcDNA were purchased from GenePharma (Shanghai, China). Lipofectamine 3000 reagent (Invitrogen, Carlsbad, CA, U.S.A.) was utilized for transfection.

### MTT assay

HaCaT cells were seeded into 96-well plates to settle down. The next day, at appropriate time points, 20 μl 3-(4,5-Dimethylthiazol-2-yl)-2,5-diphenyltetrazolium bromide (MTT) (5 mg/ml; Sigma, St. Louis, MO, U.S.A.) was added to incubate with HaCaT cells for 4 h. The optical density at 490 nm was examined using the microplate spectrophotometer (Bio-Tek Instruments, Winooski, VT, U.S.A.).

### Transwell assays

After transfection for 24 h, approximately 3 × 10^4^ HaCaT cells in serum-free medium were plated into the 8 μM upper chambers (Costar, Corning, NY, U.S.A.) pre-coated with (transwell invasion assay) or without (transwell migration assay) diluted Matrigel solution (BD Biosciences, Franklin Lakes, NJ, U.S.A.). A total of 600 μl culture medium plus 10% FBS was added to the lower chambers to fill them. After 24-h of incubation, HaCaT cells remained on the upper surface of the membrane (un-migrated or un-invaded cells) were mechanically discarded. HaCaT cells migrated or invaded into the lower surface of the membrane were fixed and stained by Crystal Violet (Sangon Biotech). The number of HaCaT cells in five random fields with the magnification of 100 times was counted.

### Western blot assay

Total proteins were isolated using Radioimmunoprecipitation assay (RIPA) lysis buffer (Sangon Biotech), and protein concentrations were determined by the BCA Kit (Beyotime, Haimen, China). A total of 30 μg protein/line was separated by the 12% sodium dodecyl sulfate/polyacrylamide gel electrophoresis (SDS/PAGE) gel followed by transferred to the polyvinylidene fluoride (PVDF) membrane (Bio-Rad). The membrane was sealed by the 5% skimmed milk for 1 h, and primary antibodies, including anti-Ki67 (ab92742; Abcam, Cambridge, MA, U.S.A.), anti-matrix metallopeptidase 9 (anti-MMP9, ab76003; Abcam), anti-GAB1 (ab133486; Abcam) and anti-GAPDH (ab8245; Abcam), were incubated with the membrane overnight at 4°C. After 2-h incubation with the horseradish peroxidase (HRP)-labeled secondary antibody (Abcam), protein signals were visualized by the enhanced chemiluminescence (ECL) kit (Pierce Biotechnology, Rockford, IL, U.S.A.). Image Lab analysis software (Version 4.0; Bio-Rad) was used to assess the fold change of proteins.

### Dual-luciferase reporter assay

Bioinformatics software (StarBase) was used to predict the targets of circ_0061012 and miR-194-5p. The fragment of circ_0061012 or the 3′ untranslated region (3′UTR) of GAB1, including the binding sites with miR-194-5p, along with these fragments with mutant miR-194-5p-binding sites, were cloned into pmirGLO (Promega, Madison, WI, U.S.A.). HaCaT cells were seeded into 24-well plates. The next day, these cells were co-transfected with luciferase reporter plasmids and miR-194-5p or miR-NC. After 48 h of transfection, luciferase activity was determined with the Dual-Luciferase Reporter Assay System (Promega).

### RNA immune co-precipitation assay

HaCaT cells were lysed and cell lysates were incubated with protein-A Sepharose beads (Bio-Rad) which pre-coated with Argonaute-2 (Ago2) antibody (ab32381; Abcam) or Immunoglobulin G (IgG) antibody for 3 h. The complexes were enriched by protein-A Sepharose beads. After isolating with TRIzol reagent (Thermo Fisher Scientific), qRT-PCR was implemented for the determination of RNA expression.

### Statistical analysis

Statistical analysis of three independent experiments with at least three technical repetitions was conducted using GraphPad Prism 7.0 (GraphPad, La Jolla, CA, U.S.A.), and the statistical data were expressed as mean ± standard deviation. Differences were evaluated by Student’s *t*-test (two groups) or one-way analysis of variance (ANOVA) followed by Tukey’s test (multiple groups). Correlation analysis was conducted using Spearman’s correlation analysis test. *P*-value less than 0.05 was considered as statistically significant.

## Results

### Circ_0061012 is highly expressed in the lesional skin of psoriasis patients and IL-22-induced HaCaT cells

**Figure 1:**
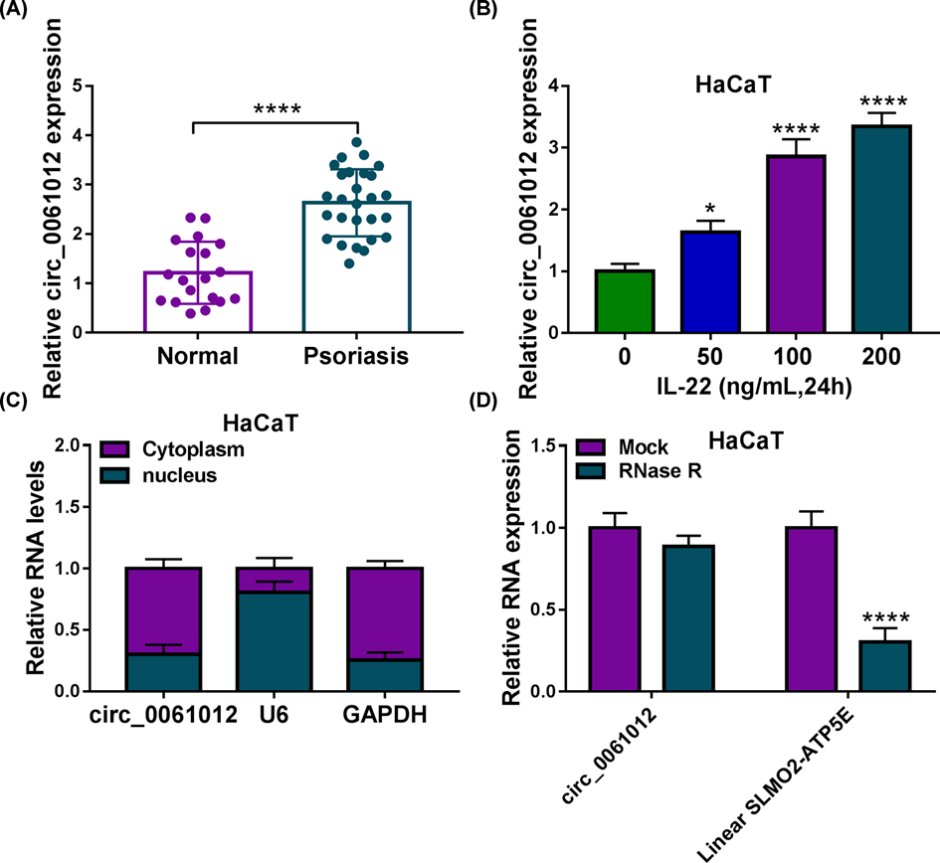
Circ_0061012 is highly expressed in the lesional skin of psoriasis patients and IL-22-induced HaCaT cells (**A**) The expression of circ_0061012 was examined in the lesional skin of psoriasis patients (*n*=27) and healthy skin of normal volunteers (*n*=27) by qRT-PCR. (**B**) The level of circ_0061012 was determined in HaCaT cells induced by IL-22 of 50, 100 or 200 ng/ml for 24 h via qRT-PCR. (**C**) The proportion of circ_0061012 distribution in cytoplasmic fraction or nuclear fraction was analyzed. U6 and GAPDH were utilized as the nuclear marker and cytoplasmic marker, respectively. (**D**) Exonuclease RNase R was utilized to assess if circ_0061012 was a circular transcript. RNA samples isolated from HaCaT cells were digested with RNase R or not, and the levels of circ_0061012 and its linear counterpart were measured by qRT-PCR. The experiments were independently repeated for three times with at least three technical repetitions. *P<0.05, ****P<0.0001.

The up-regulated expression of circ_0061012 was observed in the lesional skin tissues of psoriasis patients (*n*=27) relative to healthy skin tissues of normal volunteers (*n*=27) (Figure 1A). Meanwhile, circ_0061012 expression was significantly enhanced in HaCaT cells upon IL-22 treatment in a dose-dependent manner as shown in Figure 1B. Prior to investigate the contribution of circ_0061012 in psoriasis progression, we explored its subcellular distribution in HaCaT cells. U6 acted as nuclear marker while GAPDH was used as cytoplasmic marker. As shown in Figure 1C, circ_0061012 was mainly distributed in the cytoplasm of HaCaT cells. RNA samples isolated from HaCaT cells were divided into two equal parts, and these two parts were treated with RNase R nor not. The expression of circ_0061012 remained unaffected while the level of its linear form was notably reduced after RNase R digestion (Figure 1D). Overall, circ_0061012 was highly expressed in psoriasis patients and it was mainly distributed in the cytoplasmic fraction of HaCaT cells.

### IL-22 promotes cell proliferation, migration and invasion of HaCaT cells through up-regulating circ_0061012

**Figure 2:**
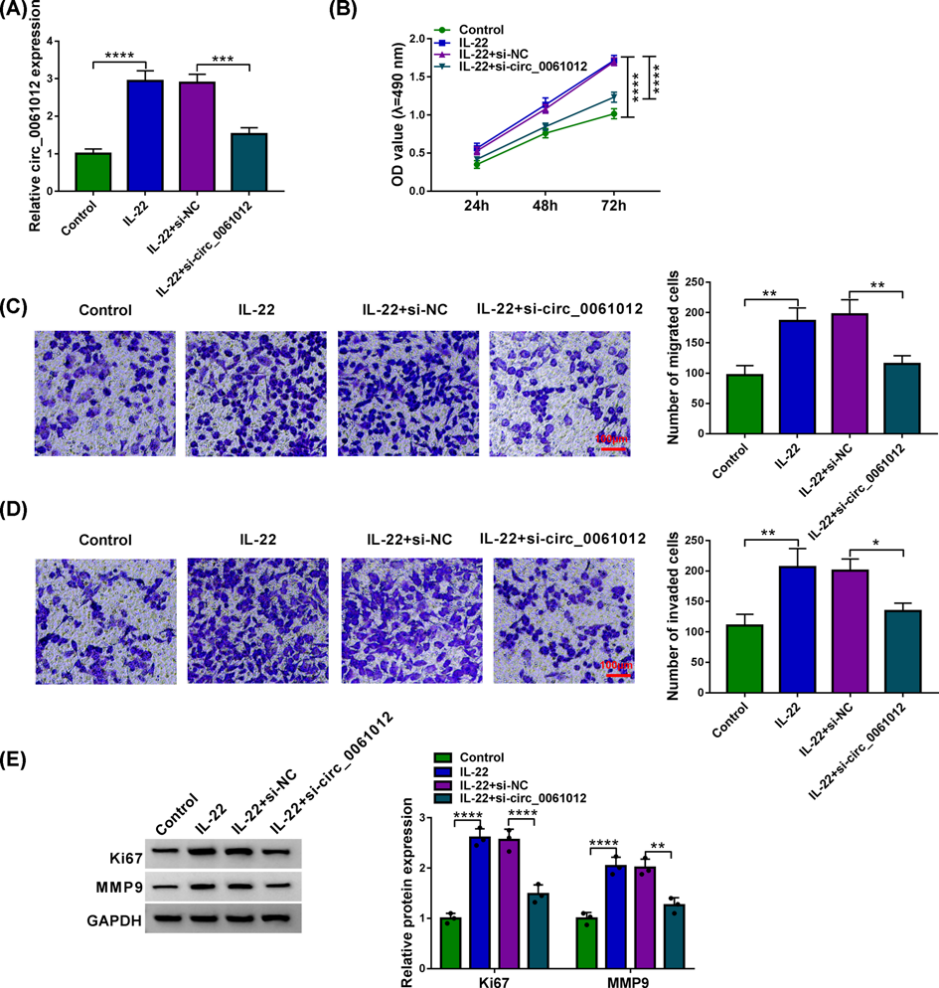
IL-22 promotes cell proliferation, migration and invasion of HaCaT cells through up-regulating circ_0061012 (**A**–**E**) HaCaT cells were divided into Control group, IL-22 treatment group, IL-22 + si-NC group and IL-22 + si-circ_0061012 group. (A) The expression of circ_0061012 was determined via qRT-PCR. (B) Cell viability of HaCaT cells at specific time points in four groups was measured by MTT assay, and cell proliferation curve was plotted to analyze cell proliferation ability. (C) Transwell migration assay was performed to analyze cell migration ability. Migrated HaCaT cells were stained by Crystal Violet, and the number of migrated cells in five random fields was counted. Scale bar = 100 μm. (D) The invasion capacity of HaCaT cells was assessed via transwell invasion assay. Invaded HaCaT cells were dyed by Crystal Violet, and the number of invaded cells in five random fields was analyzed. Scale bar = 100 μm. (**E**) Western blot assay was conducted to measure the expression of proliferation marker (Ki67) and metastasis marker (MMP9) in HaCaT cells. The experiments were independently repeated for three times with at least three technical repetitions. *P<0.05, **P<0.01, ***P<0.001, ****P<0.0001.

To investigate the significance of IL-22-induced up-regulation of circ_0061012 in HaCaT cells, we silenced circ_0061012 in IL-22-induced HaCaT cells. IL-22 stimulation enhanced the expression of circ_0061012, and this effect was partly overturned by the addition of si-circ_0061012 (Figure 2A). IL-22 treatment promoted the proliferation of HaCaT cells via MTT assay compared with Control group, and circ_0061012 knockdown counteracted this promoting effect in the proliferation of HaCaT cells (Figure 2B). Transwell assays were performed to explore if IL-22 functioned in the migration and invasion of HaCaT cells through elevating circ_0061012 expression. The number of migrated or invaded HaCaT cells was elevated with IL-22 treatment, and the addition of si-circ_0061012 overturned IL-22-mediated promoting effects in the migration and invasion of HaCaT cells (Figure 2C,D). The expression of proliferation marker Ki67 and metastasis marker MMP9 was measured by Western blot assay to verify the influences of circ_0061012 silencing in IL-22-stimulated HaCaT cells. IL-22 induced promoting effects in the levels of Ki67 and MMP9 were largely alleviated by the introduction of si-circ_0061012 in HaCaT cells (Figure 2E). These results suggested that IL-22 promoted proliferation and motility of keratinocytes partly through elevating circ_0061012 expression in psoriasis.

### Circ_0061012 interacts with miR-194-5p in HaCaT cells

**Figure 3:**
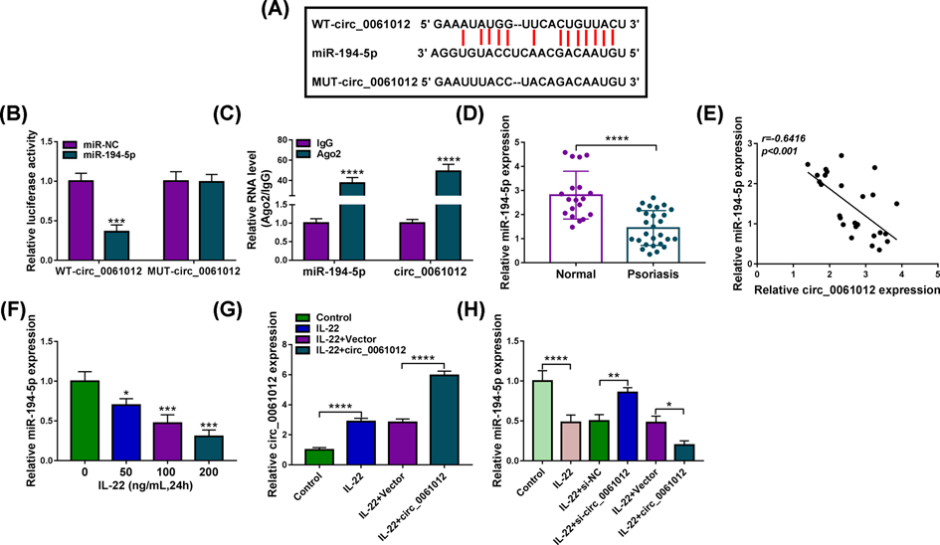
Circ_0061012 interacts with miR-194-5p in HaCaT cells (**A**) The possible miRNA targets of circ_0061012 were predicted by StarBase software, and the putative binding sites between miR-194-5p and circ_0061012 were shown. The mutant miR-194-5p-binding sites in circ_0061012 were also shown. (**B**) Dual-luciferase reporter assay was implemented to test the interaction between miR-194-5p and circ_0061012. Luciferase plasmid (WT-circ_0061012 or MUT-circ_0061012) and miR-194-5p or miR-NC were co-transfected into HaCaT cells, and the luciferase intensities in four groups were determined after transfection for 48 h. (**C**) RIP assay was used to confirm the interaction between miR-194-5p and circ_0061012. Ago2 antibody was used to pull down miRNA-contained RNA-induced silencing complex (RISC) to test if there was spatial interaction between miR-194-5p and circ_0061012 in RISC. (**D**) The expression of miR-194-5p in healthy skin tissues (*n*=27) and psoriasis skin tissues (*n*=27) was analyzed by qRT-PCR. (**E**) Linear correlation between the expression of miR-194-5p and circ_0061012 was analyzed by Spearman’s correlation coefficient. (**F**) The level of miR-194-5p was determined in HaCaT cells treated with different doses of IL-22 (50, 100 or 200 ng/ml) by 24 h via qRT-PCR. (**G**) HaCaT cells transfected with circ_0061012 or Vector were subsequently exposed to 100 ng/ml IL-22 for 24 h, and the expression of circ_0061012 was examined by qRT-PCR. (**H**) qRT-PCR was implemented to measure the expression of miR-194-5p in Control group, IL-22 group, IL-22 + si-NC group, IL-22 + si-circ_0061012 group, IL-22 + Vector group and IL-22 + circ_0061012 group. The experiments were independently repeated for three times with at least three technical repetitions. *P<0.05, **P<0.01, ***P<0.001, ****P<0.0001.

StarBase database was used to predict the targets of circ_0061012, and miR-194-5p was predicted as a possible target of circ_0061012. The complementary sequence between circ_0061012 and miR-194-5p was listed in Figure 3A. To validate the hypothesis that miR-194-5p interacted with circ_0061012, we conducted dual-luciferase reporter assay and RNA immune co-precipitation (RIP) assay. MiR-194-5p accumulation resulted in a significant reduction in luciferase activity in WT-circ_0061012 group compared with miR-NC group (Figure 3B), suggested that miR-194-5p was a target of circ_0061012 in HaCaT cells. Meanwhile, compared with miR-NC and MUT-circ_0061012 group, luciferase activity was unaffected in miR-194-5p and MUT-circ_0061012 group (Figure 3B), suggested that circ_0061012 interacted with miR-194-5p via the complementary sites shown in Figure 3A. Compared with IgG group, both miR-194-5p and circ_0061012 were pulled-down in Ago2 group (Figure 3C), demonstrating the interaction between miR-194-5p and circ_0061012. There was a notable down-regulation in miR-194-5p expression in psoriasis lesional tissues relative to healthy skin tissues (Figure 3D). MiR-194-5p expression was negatively correlated with the level of circ_0061012 (Figure 3E). With the increased dose of IL-22, miR-194-5p expression was gradually decreased in HaCaT cells (Figure 3F). IL-22-induced up-regulation in circ_0061012 expression was further enhanced with the addition of circ_0061012 overexpression plasmid in HaCaT cells (Figure 3G). IL-22 stimulation decreased the abundance of miR-194-5p, and its expression was partly recovered in IL-22 and si-circ_0061012 group (Figure 3H). Besides, IL-22-induced down-regulation in miR-194-5p expression was further potentiated with the addition of circ_0061012 in HaCaT cells (Figure 3H). These findings suggested that miR-194-5p was a target of circ_0061012, and miR-194-5p was negatively regulated by circ_0061012 in HaCaT cells.

### Circ_0061012 silencing-mediated effects are partly overturned by the addition of anti-miR-194-5p in HaCaT cells upon IL-22 treatment

**Figure 4:**
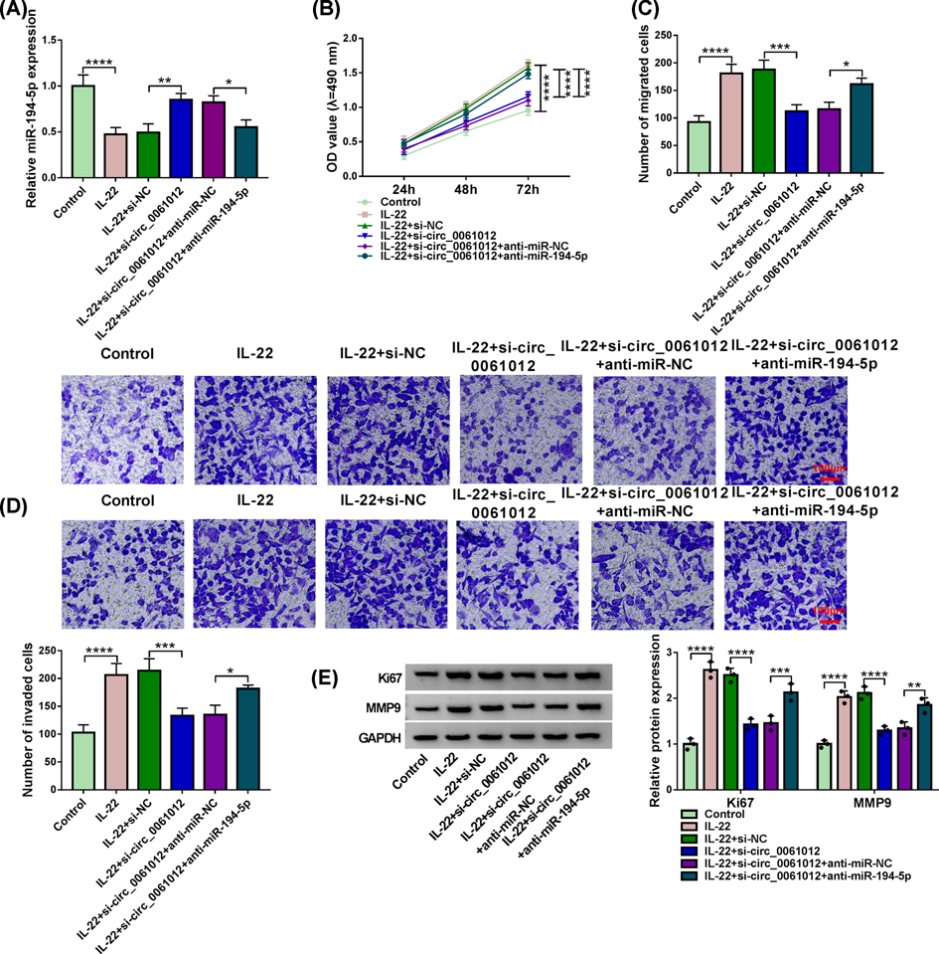
Circ_0061012 silencing-mediated effects are partly overturned by the addition of anti-miR-194-5p in HaCaT cells upon IL-22 treatment (**A**–**E**) HaCaT cells were transfected with si-NC, si-circ_0061012, si-circ_0061012 + anti-miR-NC or si-circ_0061012 + anti-miR-194-5p prior to 100 ng/ml IL-22 stimulation for 24 h. Untreated HaCaT cells were used as Control group. (A) MiR-194-5p expression was examined in HaCaT cells by qRT-PCR. (B) MTT assay was conducted to measure cell viability at specific time points. Cell proliferation curve was generated to analyze cell proliferation ability in four groups. (C,D) Transwell assays were used to analyze cell migration and invasion abilities through counting the numbers of migrated and invaded HaCaT cells in five random fields. HaCaT cells were stained using crystal violet. Scale bar = 100 μm. (E) The expression of proliferation-related marker Ki67 and metastasis-related marker MMP9 was examined in HaCaT cells by Western blot assay. The experiments were independently repeated for three times with at least three technical repetitions. *P<0.05, **P<0.01, ***P<0.001, ****P<0.0001.

Si-circ_0061012 and anti-miR-194-5p were co-transfected into HaCaT cells upon IL-22 stimulation to investigate if circ_0061012 exerted its function through targeting miR-194-5p. Circ_0061012 knockdown rescued the expression of miR-194-5p which was down-regulated by the treatment of IL-22 (Figure 4A). Furthermore, the expression of miR-194-5p was reduced with the addition of anti-miR-194-5p once again (Figure 4A). Circ_0061012 silencing-mediated suppression in cell proliferation was attenuated by the introduction of anti-miR-194-5p in IL-22-treated HaCaT cells (Figure 4B). MiR-194-5p knockdown also recovered the migration and invasion abilities which were inhibited by silencing of circ_0061012 in HaCaT cells upon IL-22 treatment (Figure 4C,D). The protein expression of Ki67 and MMP9 was both reduced by the silencing of circ_0061012 in IL-22-stimulated HaCaT cells, and the levels of these two proteins were largely recovered in IL-22 + si-circ_0061012 + anti-miR-194-5p group (Figure 4E). Taken together, circ_0061012 silencing suppressed IL-22-induced proliferation and metastasis of keratinocytes through up-regulating miR-194-5p in psoriasis *in vitro*.

### MiR-194-5p interacts with the 3′UTR of GAB1 in HaCaT cells

**Figure 5:**
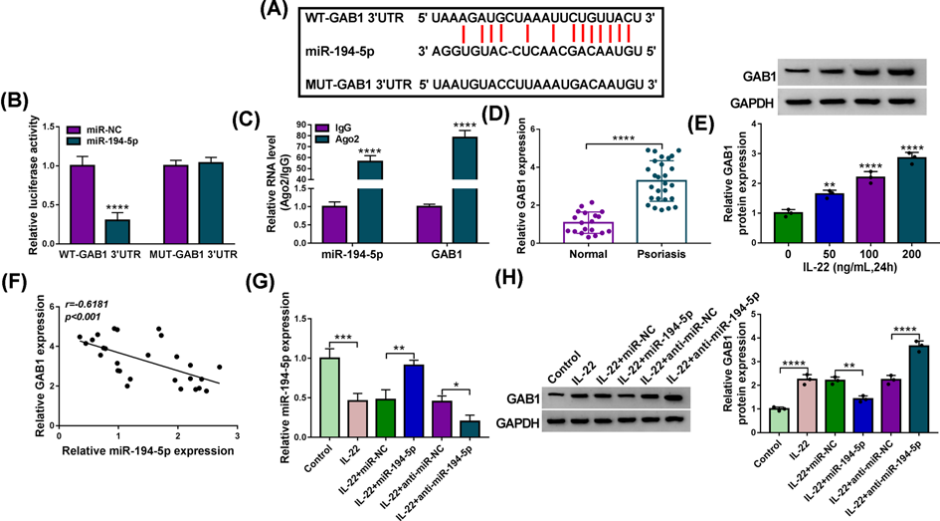
MiR-194-5p interacts with the 3′UTR of GAB1 in HaCaT cells (**A**) GAB1 was predicted to be a possible target of miR-194-5p via StarBase software, and their putative binding sequences were shown. The mutant miR-194-5p-binding sites in GAB1 were also shown. (**B**) The target interaction between miR-194-5p and GAB1 was verified by dual-luciferase reporter assay. HaCaT cells were co-transfected with luciferase plasmid (WT-GAB1 3′UTR or MUT-GAB1 3′UTR) and miR-194-5p or miR-NC for 48 h, and luciferase activities were examined. (**C**) RIP assay was carried out to confirm the interaction between miR-194-5p and GAB1. (**D**) The mRNA expression of GAB1 in 27 pairs of normal skin tissues and psoriasis lesional skin tissues was measured by qRT-PCR. (**E**) Western blot assay was performed to detect the protein level of GAB1 in HaCaT cells stimulated with 50, 100 or 200 ng/ml IL-22 for 24 h. (**F**) Spearman’s correlation coefficient was used to assess the linear correlation between the expression of miR-194-5p and GAB1. (**G,H**) The expression of miR-194-5p and GAB1 protein was examined in HaCaT cells in Control group, IL-22 group, IL-22 + miR-NC group, IL-22 + miR-194-5p group, IL-22 + anti-miR-NC group and IL-22 + anti-miR-194-5p group via qRT-PCR or Western blot assay, respectively. The experiments were independently repeated for three times with at least three technical repetitions. *P<0.05, **P<0.01, ***P<0.001, ****P<0.0001.

GAB1 was predicted as a target of miR-194-5p by StarBase software (Figure 5A). HaCaT cells were co-transfected with miR-NC or miR-194-5p and WT-GAB1 3′UTR or MUT-GAB1 3′UTR. Luciferase activity was prominently reduced in miR-194-5p and WT-GAB1 3′UTR group rather than miR-194-5p and MUT-GAB1 3′UTR group (Figure 5B), suggesting the interaction between miR-194-5p and GAB1 in HaCaT cells. Meanwhile, miR-194-5p and GAB1 were both enriched in Ago2 group relative to IgG group (Figure 5C), which further verified the target interaction between miR-194-5p and GAB1 in HaCaT cells. GAB1 mRNA level was prominently enhanced in skin tissues of psoriasis patients when compared with healthy volunteers (Figure 5D). The results of Western blot assay revealed that GAB1 protein level was gradually up-regulated with the increased concentration of IL-22 in HaCaT cells (Figure 5E). There was a negative correlation between the expression of miR-194-5p and GAB1 (Figure 5F). MiR-194-5p overexpression attenuated the suppressive influence of IL-22 treatment in the level of miR-194-5p, and IL-22-mediated down-regulation in miR-194-5p expression was further aggravated with the addition of anti-miR-194-5p in HaCaT cells (Figure 5G). MiR-194-5p mimics addition attenuated IL-22-induced promoting effect in GAB1 expression, while the addition of anti-miR-194-5p further potentiated IL-22-induced up-regulation of GAB1 expression in HaCaT cells (Figure 5H). Taken together, GAB1 was a target of miR-194-5p in HaCaT cells.

### MiR-194-5p suppresses IL-22-induced progression of psoriasis partly through targeting GAB1 in HaCaT cells

**Figure 6:**
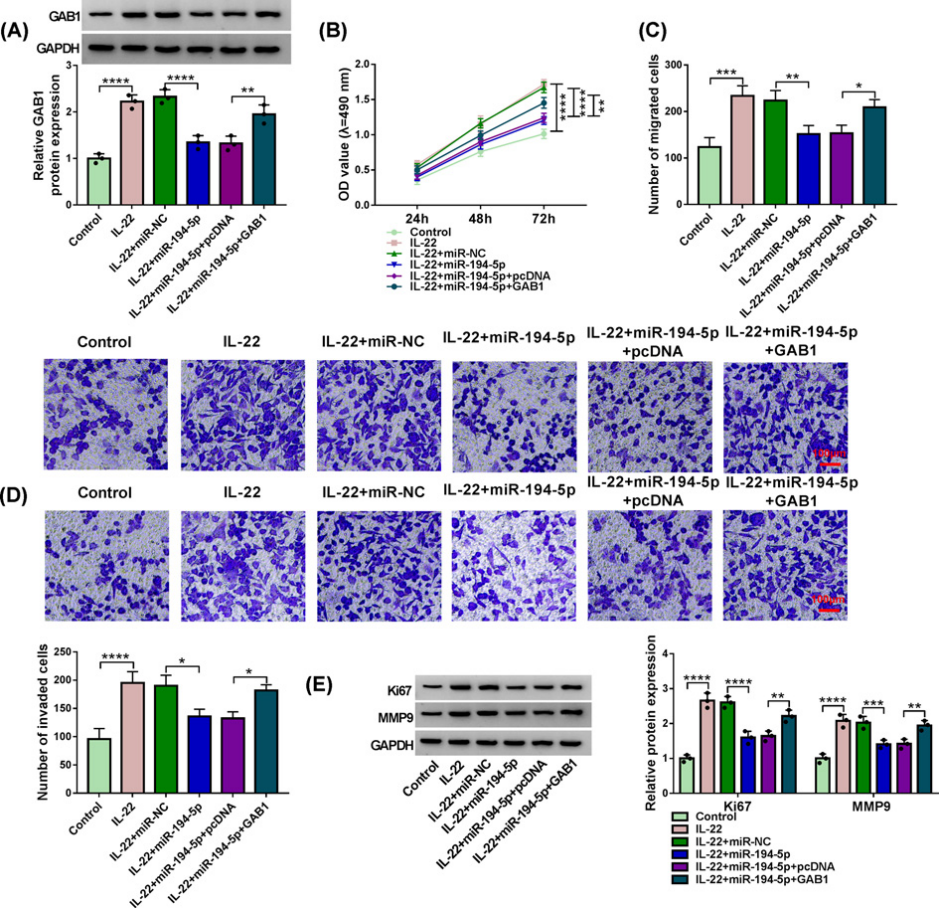
MiR-194-5p suppresses IL-22-induced progression of psoriasis partly through targeting GAB1 in HaCaT cells (**A**–**E**) HaCaT cells transfected with miR-NC, miR-194-5p, miR-194-5p + pcDNA or miR-194-5p + GAB1 were treated with 100 ng/ml IL-22 for 24 h. (A) Western blot assay was utilized to measure the protein level of GAB1 in HaCaT cells. (B) MTT assay was used to evaluate cell viability of HaCaT cells at specific time points, and cell proliferation curve was plotted to evaluate cell proliferation capacity. (C,D) Transwell migration and invasion assays were used to measure the abilities of migration and invasion of HaCaT cells. The numbers of migrated and invaded HaCaT cells (stained by Crystal Violet) in five random fields were counted. Scale bar = 100 μm. (E) Western blot assay was used to measure the levels of Ki67 and MMP9 in HaCaT cells. The experiments were independently repeated for three times with at least three technical repetitions. *P<0.05, **P<0.01, ***P<0.001, ****P<0.0001.

MiR-194-5p and GAB1 plasmid were co-transfected into IL-22-induced HaCaT cells to explore if miR-194-5p suppressed psoriasis progression via targeting and suppressing GAB1. MiR-194-5p overexpression reduced the expression of GAB1 protein, which was up-regulated with IL-22 treatment (Figure 6A). The expression of GAB1 protein was recovered with the addition of GAB1 plasmid in IL-22 + miR-194-5p + GAB1 group (Figure 6A). MiR-194-5p-mediated suppressive influence in cell proliferation in IL-22-induced HaCaT cells was partly counteracted by the addition of GAB1 plasmid (Figure 6B). MiR-194-5p overexpression restrained the migration and invasion of HaCaT cells upon IL-22 stimulation, while the metastasis ability was partly rescued with the addition of GAB1 plasmid (Figure 6C,D). MiR-194-5p accumulation reduced the protein expression of Ki67 and MMP9 in IL-22-induced HaCaT cells, and the levels of Ki67 and MMP9 were largely recovered with the introduction of GAB1 plasmid (Figure 6E). These results demonstrated that miR-194-5p suppressed psoriasis progression through inhibiting the proliferation, migration and invasion of HaCaT cells via targeting GAB1.

### Circ_0061012 up-regulates the level of GAB1 through sponging miR-194-5p in IL-22-induced HaCaT cells

**Figure 7:**
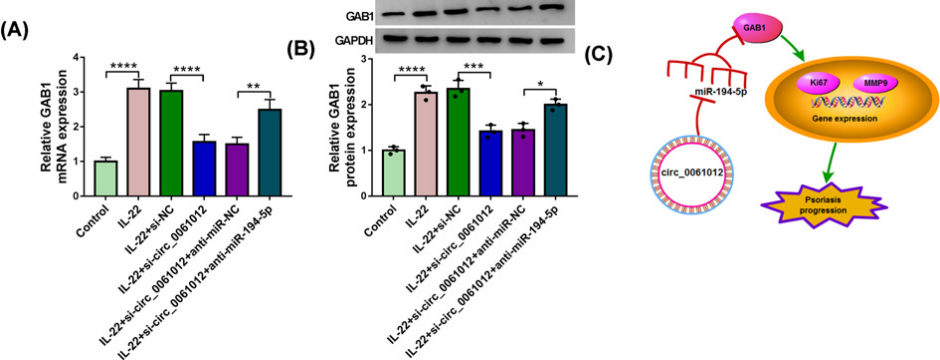
Circ_0061012 up-regulates the level of GAB1 through sponging miR-194-5p in IL-22-induced HaCaT cells (**A,B**) The mRNA and protein levels of GAB1 were measured in HaCaT cells treated with IL-22, IL-22 + si-NC, IL-22 + si-circ_0061012, IL-22 + si-circ_0061012 + anti-miR-NC or IL-22 + si-circ_0061012 + anti-miR-194-5p by qRT-PCR and Western blot assay. (**C**) The diagram revealed that circ_0061012 accelerated psoriasis progression through miR-194-5p/GAB1 axis. The experiments were independently repeated for three times with at least three technical repetitions. *P<0.05, **P<0.01, ***P<0.001, ****P<0.0001.

Given the negative regulatory relationship between miR-194-5p and circ_0061012 or GAB1, we subsequently aimed to investigate the modulatory relationship between circ_0061012 and GAB1. HaCaT cells were transfected with si-NC, si-circ_0061012, si-circ_0061012 + anti-miR-NC or si-circ_0061012 + anti-miR-194-5p prior to IL-22 stimulation. The mRNA and protein abundance of GAB1 was enhanced with IL-22 treatment, and this effect was attenuated with the silencing of circ_0061012 (Figure 7A,B). Circ_0061012 silencing-mediated reduction in the mRNA and protein levels of GAB1 was counteracted by the addition of anti-miR-194-5p (Figure 7A,B), suggested that circ_0061012 enhanced the abundance of GAB1 mRNA and protein through sponging miR-194-5p in HaCaT cells upon IL-22 treatment. As shown in Figure 7C, we concluded that circ_0061012 elevated the expression of Ki67 and MMP9 to promote psoriasis progression through targeting miR-194-5p/GAB1 axis.

## Discussion

We explored the biological contribution of circ_0061012 in psoriasis progression. Circ_0061012 expression was notably enhanced in the lesional samples relative to healthy skin specimens. Loss-of-function experiments demonstrated that IL-22 accelerated the proliferation and metastasis of HaCaT cells partly through up-regulating circ_0061012. Subsequently, circ_0061012/miR-194-5p/GAB1 signal axis was identified to be involved in the pathogenesis of psoriasis. Our study discovered for the first time the crucial role of circ_0061012 in the proliferation and metastasis of keratinocytes.

CircRNAs modulate the progression of human diseases. For instance, circ-BANP accelerated the development of lung cancer through targeting miR-503/LARP1 axis [20]. Circ_0000950 accelerated neuron apoptosis, inhibited the outgrowth of neurite and enhanced the levels of inflammation-related cytokines via miR-103/PTGS2 axis in Alzheimer’s disease [21]. A large number of circRNAs were dysregulated in psoriasis specimens compared with healthy skin specimens, and circ_0061012 was one of the circRNAs that were up-regulated in psoriasis [11]. Consistent with former article [11], we found that circ_0061012 expression was elevated in psoriasis lesional specimens compared with healthy specimens. Also, circ_0061012 level was increased in IL-22-induced HaCaT cells in a dose-dependent manner. IL-22 stimulation promoted cell proliferation, migration and invasion of HaCaT cells, and these effects were largely attenuated by the silencing of circ_0061012, demonstrated that IL-22 promoted psoriasis progression partly through enhancing circ_0061012 expression.

MiRNAs are involved in the regulation of psoriasis progression [12,13]. Xu et al. found that miR-155 contributed to the proliferation and suppressed cell apoptosis through regulating PTEN signal pathway in psoriasis [22]. Tang et al. demonstrated that the down-regulation of miR-187 accelerated the proliferation of keratinocytes in psoriasis [23]. MiR-194-5p was predicted to be a binding partner of circ_0061012 via StarBase database, and this interaction was confirmed by dual-luciferase reporter assay and RIP assay. MiR-194-5p restrained the malignant behaviors of many cancer cells [24–26]. Wang et al. claimed that miR-194-5p suppressed the motility of bladder cancer cells through E2F3 [24]. MiR-194 was reported to suppress cell proliferation and induce cell differentiation of keratinocytes via GRHL2 [16]. MiR-194-5p expression was declined in psoriasis specimens relative to healthy skin specimens. Furthermore, IL-22 treatment dose-dependently decreased the level of miR-194-5p in HaCaT cells. Compensation experiments revealed that circ_0061012 silencing restrained IL-22-induced proliferation and metastasis in HaCaT cells through targeting miR-194-5p.

The interaction between miR-194-5p and GAB1 was verified by dual-luciferase reporter assay and RIP assay. GAB1 facilitated the inflammation and fibrosis in systemic sclerosis [27]. GAB1 was abnormally expressed in sclerosis and psoriasis [28]. GAB1 contributed to the proliferation and differentiation of epidermal cells [17]. GAB1 expression was aberrantly up-regulated in lesional skin tissues of psoriasis patients and IL-22-induced HaCaT cells compared with that in healthy skin tissues and un-treated HaCaT cells. Rescue experiments suggested that miR-194-5p restrained proliferation and motility of HaCaT cells upon IL-22 treatment through targeting GAB1. Circ_0061012 acted as the molecular sponge of miR-194-5p to elevate the expression of GAB1 in HaCaT cells.

In conclusion, circ_0061012 contributed to IL-22-induced proliferation and motility of HaCaT cells through targeting miR-194-5p/GAB1 axis, providing a novel insight into developing new therapeutic targets for psoriasis patients.

## Data Availability

The datasets used and/or analyzed during the current study are available from the corresponding author on reasonable request.

## Abbreviations

Ago2: Argonaute-2
circRNA: circular RNA
circ_0061012: circular RNA 0061012
FBS: fetal bovine serum
GAB1: GRB2 associated binding protein 1
GAPDH: glyceraldehyde-3-phosphate dehydrogenase
IgG: immunoglobulin G
IL-22: interleukin-22
miRNA: microRNA
MMP9: matrix metallopeptidase 9
mRNA: messenger RNA
MTT: 3-(4,5-Dimethylthiazol-2-yl)-2,5-diphenyltetrazolium bromide
qRT-PCR: quantitative real-time polymerase chain reaction
RIP: RNA immune co-precipitation
3′UTR: 3′ untranslated region
